# cN+pN0 disease does not portend a less favorable prognosis compared with cN0pN0 in patients with resected oral cavity squamous cell carcinoma

**DOI:** 10.1002/cam4.4187

**Published:** 2021-09-23

**Authors:** Chien‐Yu Lin, Li‐Yu Lee, Nai‐Ming Cheng, Shu Ru Lee, Chi‐Ying Tsai, Chuen Hsueh, Kang‐Hsing Fan, Hung‐Ming Wang, Chia‐Hsun Hsieh, Shu‐Hang Ng, Chih‐Hua Yeh, Chih‐Hung Lin, Chung‐Kan Tsao, Tuan‐Jen Fang, Shiang‐Fu Huang, Li‐Ang Lee, Chung‐Jan Kang, Ku‐Hao Fang, Yu‐Chien Wang, Wan‐Ni Lin, Li‐Jen Hsin, Tzu‐Chen Yen, Chun‐Ta Liao

**Affiliations:** ^1^ Department of Radiation Oncology Chang Gung Memorial Hospital and Chang Gung University Taoyuan Taiwan, ROC; ^2^ Department of Pathology Chang Gung Memorial Hospital and Chang Gung University Taoyuan Taiwan, ROC; ^3^ Department of Nuclear Medicine and Molecular Imaging Center Chang Gung Memorial Hospital and Chang Gung University Taoyuan Taiwan, ROC; ^4^ Research Service Center for Health Information Chang Gung University Taoyuan Taiwan, ROC; ^5^ Department of Oral and Maxillofacial Surgery Chang Gung Memorial Hospital Chang Gung University Taoyuan Taiwan, ROC; ^6^ Department of Medical Oncology Chang Gung Memorial Hospital and Chang Gung University Taoyuan Taiwan, ROC; ^7^ Department of Diagnostic Radiology Chang Gung Memorial Hospital and Chang Gung University Taoyuan Taiwan, ROC; ^8^ Department of Plastic and Reconstructive Surgery Chang Gung Memorial Hospital and Chang Gung University Taoyuan Taiwan, ROC; ^9^ Department of Otorhinolaryngology, Head and Neck Surgery Chang Gung Memorial Hospital and Chang Gung University Taoyuan Taiwan, ROC

**Keywords:** adjuvant therapy, clinical outcomes, oral cavity cancer, pathologically negative nodes, squamous cell carcinoma

## Abstract

**Background:**

We compared the clinical outcomes of patients with oral cavity squamous cell carcinoma (OCSCC) with cN+pN0 versus cN0pN0 disease.

**Methods:**

A total of 1309 OCSCC patients with pN0 disease were included. Of them, 1019 and 290 cases had cN0pN0 and cN+pN0 disease, respectively. For comparison purposes, we also examined 799 patients with pN+disease (cN0pN+/cN+pN+, *n* = 239/560). Subgroup analysis was performed in a propensity score‐matched cohort with cN0pN0 and cN+pN0 disease (*n* = 284 each).

**Results:**

Compared with cN0pN0, patients with cN+pN0 had a higher prevalence of the following variables: betel chewing, pT3−4, depth ≥10 mm, perineural invasion, and treatment with surgery and adjuvant therapy. The prognosis of patients with cN+pN0 (mean: 52 nodes) and cN0pN0 (mean: 39 nodes) disease was similar both in the original cohort and after propensity score matching. However, the 5‐year outcomes were more favorable for cN+pN0/cN0pN0 compared with cN0pN+/cN+pN+ (local control, 88%/88%/83%/81%; neck control, 94%/93%/82%/76%; distant metastases, 4%/3%/13%/31%; disease‐free survival, 84%/83%/68%/52%; disease‐specific survival, 92%/92%/77%/57%; overall survival, 81%/82%/59%/42%; all *p* values <0.001; cN+pN0 versus cN0pN0, all *p* values >0.05). cN+pN0 disease (vs. cN0pN0) was not significantly associated with local control, neck control, distant metastases, and survivals either in univariable or multivariable analyses.

**Conclusions:**

Despite a higher risk factor burden, the prognosis of patients with cN+pN0 disease did not differ from that of cases with cN0pN0. The higher nodal yield and the more frequent use of adjuvant therapy in cN+pN0 disease may explain the lack of significant differences in terms of neck control compared with cN0pN0 disease.

## Graphical abstract

Patients with oral cavity squamous cell carcinoma and cN+pN0 disease are characterized by a higher prevalence of adverse risk factors. However, this was not found to translate into a less favorable prognosis; therefore, the presence of cN+pN0 disease should not be considered as an adverse prognostic factor

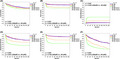

## 1 INTRODUCTION

Oral cavity squamous cell carcinoma (OCSCC)–a common type of head and neck malignancy–is the sixth most frequent cancer diagnosis made in Taiwan.^1^ Treatment is chiefly based on surgery—either with or without adjuvant therapy depending on the presence of postoperative pathological risk factors (RFs).^2^, ^3^, ^4^, ^5^, ^6^, ^7^ According to the National Comprehensive Cancer Network (NCCN) guidelines, patients presenting with extra‐nodal extension (ENE) and/or pathologic positive margins should be considered at high risk, ultimately being candidates for postoperative concurrent chemoradiation (CCRT).^8^ Cases who carry other RFs–including pT3–4 tumors, pathologically node‐positive (pN+), perineural invasion, lymphatic invasion, vascular invasion, and close margins–are deemed at intermediate risk and can be treated with either postoperative radiation therapy (RT) or CCRT.^8^ Patients with OCSCC and pathologically negative nodes (pN0) may harbor pathological RFs which can pose an indication for postoperative adjuvant therapy.^9^, ^10^, ^11^, ^12^ In the preoperative phase, cases with pN0 disease can be classified as clinically node‐negative (cN0) or node‐positive (cN+). While pathological findings remain the gold standard for tumor staging, the question as to whether the clinical outcomes of patients with cN+pN0 disease differ from those of cases with cN0pN0 remains unanswered. This issue is of practical relevance to head and neck oncologists who frequently consider patients with cN+pN0 disease as being at potential risk for neck failure. Amit et al.^13^ have previously shown that cN+pN0 disease (vs. cN0pN0) is an independent RF for 5‐year disease‐specific survival (DSS) and overall survival (OS). However, no large cohort study has thoroughly compared the clinical outcomes of patients with OCSCC and cN+pN0 versus cN0pN0−especially with respect to neck control (NC).

The purpose of this retrospective study was to conduct an extensive analysis of RFs in these two patient groups. We also examined their prognostic impact by taking into account a number of different 5‐year outcomes.

## 2 PATIENTS AND METHODS

### 2.1 Study design

**FIGURE 1:**
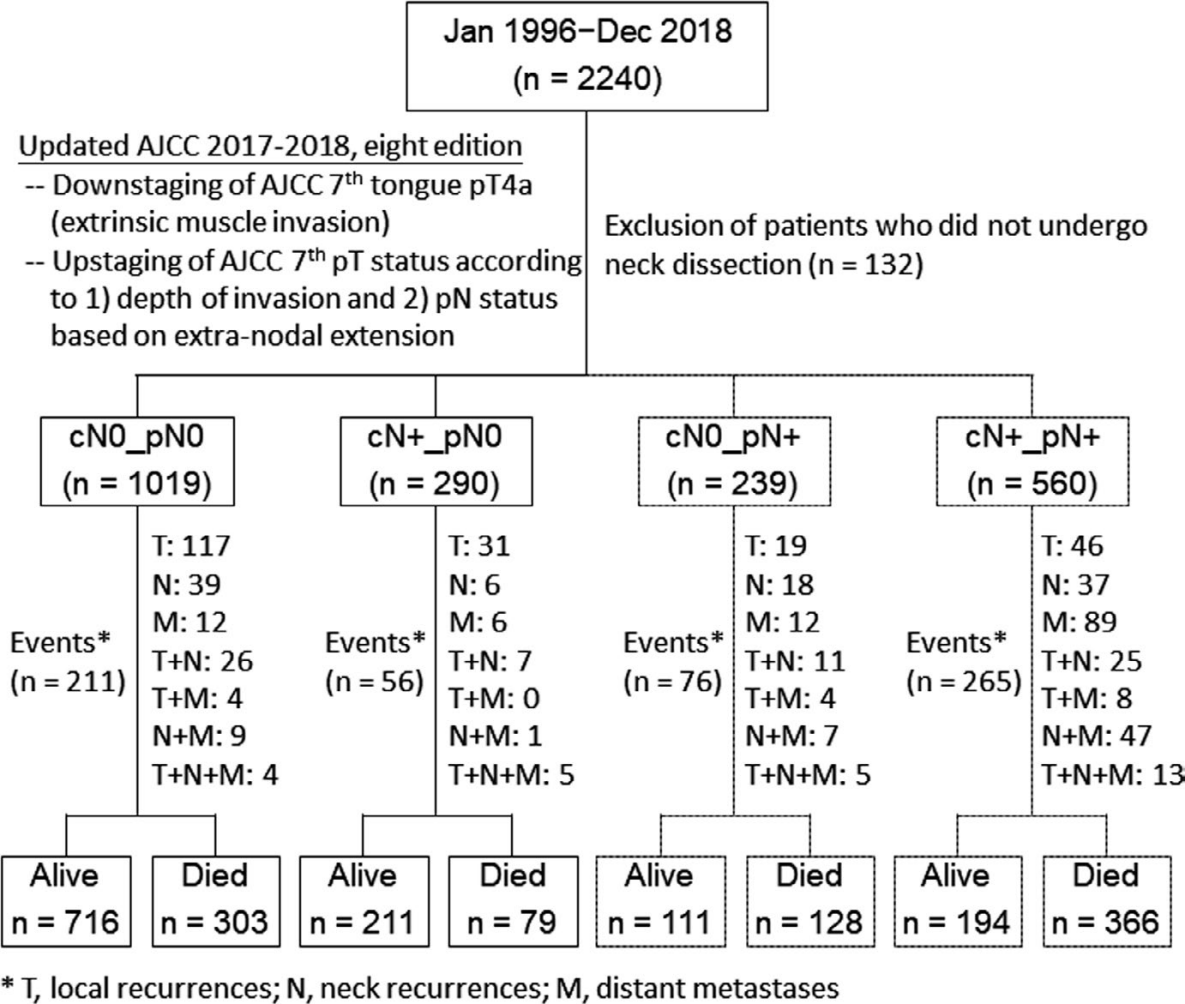
Flow of patients through the study

After obtaining appropriate institutional review board approval (CGMH 101‐4457B, 201701467B0), we retrospectively reviewed the clinical charts of all patients with first primary OCSCC who were treatment‐naïve (*n* = 2240) and consecutively referred to the Chang Gung Memorial Hospital during the period from January 1996 to December 2018. Owing to the retrospective nature of the study, the need for informed consent was waived. All cases–who were scheduled to undergo radical surgery either with (*n* = 2108) or without (*n* = 132) neck dissections (NDs)–received a thorough presurgical evaluation and staging workup as described in our previous publications.^14^, ^15^, ^16^ Clinical staging was based on the results of physical examination and imaging studies (computed tomography or magnetic resonance imaging). Patients were considered as cN+when the following criteria were met: (1) presence with at least one node with a short axis ≥1 cm, (2) identification of at least one node with a short axis <1 cm with central necrosis and/or an irregular surface, or (3) presence of a cluster of lymph nodes. In the current study, FDG‐PET imaging did not represent a criterion for diagnosing positive lymph nodes. Our institutional guidelines do not recommend fine‐needle aspiration cytology and this technique was not applied for clinical staging even in presence of suspicious regional lymph node metastases (e.g., homogeneous lymph nodes larger than 1.5−2 cm in size). Clinicopathological RFs were collected prospectively in a blinded fashion with respect to clinical endpoints. In addition, all pathological findings were independently reviewed by two experienced head‐and‐neck pathologists with a dedicated checklist. Because of the prospective collection of data on tumor depth of invasion (DOI) and ENE,^6^ disease staging was conducted according to the AJCC staging manual, eighth edition.^17^ A total of 1309 study participants were pN0, (62.1%), whereas the remaining 799 were pN+ (37.9%). Because the focus of the study was on pN0 disease (cN0pN0, *n* = 1029; cN+pN0, *n* = 290), cases with pN+disease (cN0pN+, *n* = 239; cN+pN+, *n* = 560) were included for outcome comparison purposes (Figure 1).

### 2.2 Surgery and adjuvant therapy

Primary tumors were excised with ≥1 cm margins (both peripheral and deep margins). Patients with cN+disease received level I–IV or I–V NDs, whereas cN‐ patients underwent level I–III NDs. As a general principle, patients who carried pathological RFs were treated with postoperative RT (60 Gy). RFs were classified according to the National Comprehensive Cancer Network (NCCN) guidelines until 2008^8^; subsequently, we adopted the Chang Gung Memorial Hospital (CGMH) guidelines as previously described.^7^ RT was offered to patients who carried the following pathological RFs: pT4; pT3N1; pT1–2N1 disease (N1 disease at level IV/V); 1–2 mm close margins (in the event of a second operation being unfeasible); and poor differentiation with DOI ≥4 mm. RT was also given to carriers of two minor RFs (i.e., pN1, DOI ≥10 mm, 3–4 mm close margins, poor differentiation, perineural invasion, lymphatic invasion, and vascular invasion). The radiation field was designed to include both the entire tumor bed area (with 1‐ to 2‐cm margins) and regional lymphatics. Patients who had evidence of ENE, multiple lymph node metastases, or positive margins (in the event of a second operation being unfeasible) received CCRT (66 Gy). CCRT was also administered to patients carrying at least three of the abovementioned minor RFs (pT4 and 1–2 mm close margins were considered as a single RF for CCRT).^18^, ^19^, ^20^ Chemotherapy consisted of intravenous cisplatin 50 mg/m^2^ biweekly plus daily oral tegafur 800 mg and leucovorin 60 mg, cisplatin 40 mg/m^2^ weekly, or cisplatin 100 mg/m^2^ every 3 weeks.^20^ Patients with pN0 disease who carried pathological RFs were offered postoperative RT/CCRT based on the consensus reached by our head and neck multidisciplinary team. Patients who refused the proposed approach or whose disease stage unexpectedly changed after surgery received surgery alone.

### 2.3 Statistical calculations

All participants were followed‐up for at least 24 months or until death. Patients were censored on the date of the last follow‐up (December 2020). Descriptive statistics are expressed as frequencies, percentages, means, medians, ranges, and standard deviations (SD). The study endpoints were the 5‐year rates of local control (LC), NC, distant metastasis (DM), disease‐free survival (DFS), DSS, and OS. For each endpoint, we calculated the time elapsed from the date of surgery to the date of the event of interest. Cumulative event curves were plotted using the Kaplan–Meier method and compared with the log‐rank test. The associations between RFs and the study outcomes were determined using univariate analysis (UVA) followed by multivariable Cox regression analysis (MVA). All variables included in UVA were entered as covariates in the multivariable model. Results of UVA and MVA are expressed as hazard ratios (HRs) with their 95% confidence intervals (CIs). All tests were two‐sided, and statistical significance was set as a *p* value <0.05.

## 3 RESULTS

### 3.1 Patients

#### 3.1.1 General characteristics of patients with cN0pN0 versus cN+pN0 disease

The median follow‐up time of the entire cohort study was 89 months (mean = 100 months, SD = 64 months; range = 1–283 months). The median follow‐up time for surviving patients was 106 months (mean = 117 months; SD = 62 months; range = 24–283 months).

Patients with pN0 disease were predominantly men (93.7%) and aged <65 years (85.6%). The general characteristics of patients with cN0pN0 versus cN+pN0 disease are presented in Table 1. Compared with cases showing cN0pN0 disease, those with cN+pN0 had a significantly higher prevalence of the following variables: history of betel chewing (79.8% vs. 88.3%, respectively, *p* = 0.001), pT3–4 disease (38.7% vs. 64.5%, respectively, *p* < 0.001), DOI ≥10 mm (35.3% vs. 58.8%, respectively, *p* < 0.001), perineural invasion (20.2% vs. 26.6%, respectively, *p* = 0.024), planned RT/CCRT (36.3% vs. 52.4%, respectively, *p* < 0.001), RT/CCRT given as previously planned (78.1% vs. 87.5%, respectively, *p* = 0.014), and actual treatment with adjuvant therapy (RT, 23.5% vs. 35.9%, respectively; CCRT, 4.9% vs. 10.0%, respectively, *p* < 0.001). Notably, patients with cN+pN0 disease had a higher nodal yield than those with cN0pN0 (mean, 52.1 vs. 39.4 nodes, respectively, *p* < 0.001; median, 46.0 vs. 38.0 nodes, respectively).

**TABLE 1:** General characteristics of patients with oral cavity cancer and pathologically negative nodes according to the presence or absence of clinically negative (cN0) or positive (cN+) Nodes

Characteristic (*n*, %)	Original cohort (*n* = 1309)				Propensity score‐matched cohort (*n* = 568)		
	cN0	cN+	*p*	SMD (%)	cN0	cN+	SMD (%)
	(*n* = 1019) *n* (%)	(*n* = 290) *n* (%)			(*n* = 284) *n* (%)	(*n* = 284) *n* (%)	
Sex			0.785				
Male (1227, 93.7)	956 (93.8)	271 (93.4)		1.51	273 (96.1)	270 (93.4)	5.15
Female (82, 6.3)	63 (6.2)	19 (6.6)		−1.51	11 (3.9)	14 (4.9)	−5.15
Age (years)			0.395				
<65 (1020, 85.6)	867 (85.1)	253 (87.2)		−6.25	242 (85.2)	248 (87.3)	−6.14
≥65 (189, 14.4)	152 (14.9)	37 (12.8)		6.25	42 (14.8)	36 (12.7)	6.14
Alcohol drinking			0.176				
No (418, 31.9)	335 (32.9)	83 (28.6)		9.23	83 (29.2)	82 (28.9)	0.78
Yes (891, 68.1)	684 (67.1)	207 (71.4)		−9.23	201 (70.8)	202 (71.1)	−0.78
Betel chewing			0.001				
No (240, 18.3)	206 (20.2)	34 (11.7)		23.34	32 (11.3)	34 (12.0)	−2.20
Yes (1069, 81.7)	813 (79.8)	256 (88.3)		−23.34	252 (88.7)	250 (88.0)	2.20
Cigarette smoking			0.332				
No (178, 13.6)	144 (14.1)	34 (11.7)		7.18	29 (10.2)	33 (11.6)	−4.52
Yes (1131, 86.4)	875 (85.9)	256 (88.3)		−7.18	255 (90.0)	251 (88.4)	4.52
Pathological T status			<0.001				
pT1–2 (728, 55.6)	625 (61.3)	103 (35.5)		53.48	102 (35.9)	103 (36.3)	−0.73
pT3–4 (581, 44.4)	394 (38.7)	187 (64.5)		−53.48	182 (64.1)	181 (63.7)	0.73
Differentiation			0.160				
Well/Moderate (1232, 94.1)	964 (94.6)	268 (92.4)		8.89	269 (94.7)	262 (92.3)	10.00
Poor (77, 5.9)	55 (5.4)	22 (7.6)		−8.89	15 (5.3)	22 (7.8)	−10.00
Depth of invasion*			<0.001				
<10 mm (778, 59.5)	659 (64.7)	119 (41.2)		48.74	119 (41.9)	119 (41.9)	0.00
≥10 mm (529, 40.5)	359 (35.3)	170 (58.8)		−48.21	165 (58.1)	170 (58.1)	0.00
Margin status*			0.154				
≤4 mm (133, 10.2)	97 (9.5)	36 (12.4)		−9.27	31 (10.9)	35 (12.3)	−4.40
>4 mm (1173, 89.8)	919 (90.5)	254 (87.6)		8.28	253 (89.1)	249 (87.7)	4.40
Perineural invasion*			0.024				
No (1025, 78.4)	812 (79.8)	213 (73.4)		14.77	208 (73.2)	208 (73.2)	0.00
Yes (283, 21.6)	206 (20.2)	77 (26.6)		−15.01	76 (26.8)	76 (26.8)	0.00
Lymphatic invasion*			0.424				
No (1299, 99.3)	1012 (99.4)	287 (99.0)		3.76	283 (99.7)	281 (98.9)	8.43
Yes (9, 0.7)	6 (0.6)	3 (1.0)		−4.97	1 (0.3)	3 (1.1)	−8.43
Vascular invasion*			1.000				
No (1292, 98.8)	1005 (98.7)	287 (99.0)		−3.11	278 (97.9)	281 (98.9)	−8.47
Yes (16, 1.2)	13 (1.3)	3 (1.0)		2.26	6 (2.1)	3(1.1)	8.47
Planned treatment			<0.001				
S alone (787, 60.1)	649 (63.7)	138 (47.6)		32.85			
Planned RT/CCRT (522, 39.9)	370 (36.3)	152 (52.4)	0.014	−32.85			
RT/CCRT (−) (100, 19.2)	81 (21.9)	19 (12.5)		25.08			
RT/CCRT (+) (422, 80.8)	289 (78.1)	133 (87.5)		−25.08			
Actual treatment			<0.001				
S alone (887, 67.8)	730 (71.6)	157 (54.1)		36.84	154 (54.2)	154 (54.2)	0.00
S plus RT (343, 26.2)	239 (23.5)	104 (35.9)		−27.42	104 (36.6)	102 (35.9)	1.46
S plus CCRT (79, 6.0)	50 (4.9)	29 (10.0)		−19.48	26 (9.2)	28 (9.9)	−2.40
Nodal yield							
Range (6–181)	6–146	7–181					
Mean (42.2)	39.4	52.1	<0.001				
Median (39.0)	38.0	46.0					

Abbreviations: CCRT, concurrent chemoradiotherapy; RT, radiotherapy; S, surgery; SMD, standardized mean difference.

* Unavailable data: depth of invasion (*n* = 2), margin status (*n* = 3), perineural invasion (*n* = 1), lymphatic invasion (*n* = 1), and vascular invasion (*n* = 1).

### 3.2 Five‐year outcomes of patients with cN+pN0 versus cN0pN0 disease: comparison with cN0pN+ and cN+pN+

**FIGURE 2:**
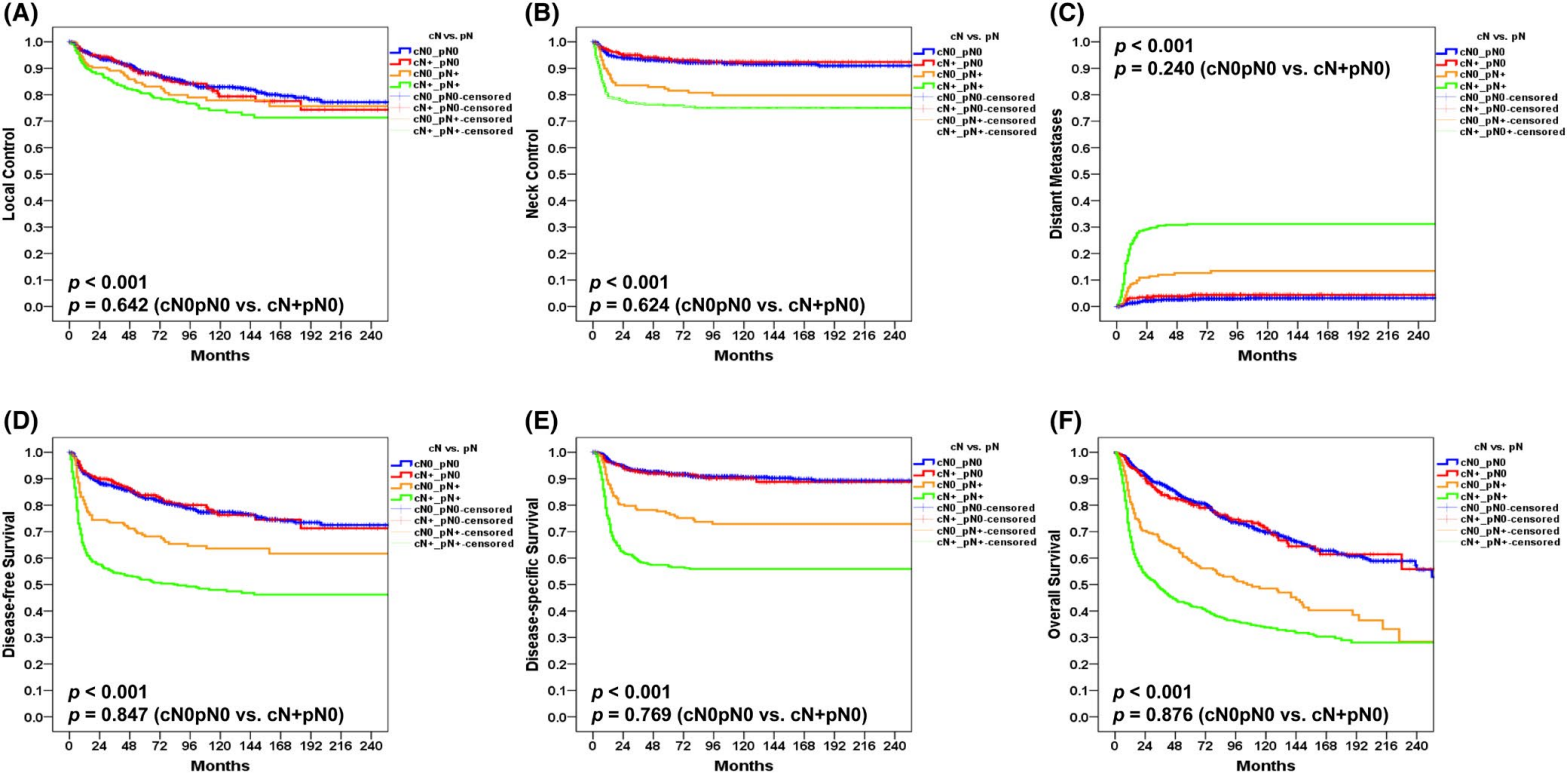
Kaplan–Meier plots of 5‐year local control (A), neck control (B), distant metastases (C), disease‐free survival (D), disease‐specific survival (E), and overall survival (F) for patients with cN0pN0, cN+pN0, cN0pN+, and cN+pN+ disease in the original data set

The 5‐year rates in patients with pN0 versus pN+disease were as follows: LC, 88% versus 81%; NC, 93% versus 78%; DM, 3% versus 25%; DFS, 83% versus 57%; DSS, 92% versus 63%; and OS, 81% versus 47%, respectively (all *p* values <0.001). The following 5‐year rates were observed in patients with cN+pN0, cN0pN0, cN0pN+, and cN+pN+ disease: LC, 88%/88%/83%/81%; NC, 94%/93%/82%/76%; DM, 4%/3%/13%/31%; DFS, 84%/83%/68%/52%; DSS, 92%/92%/77%/57%; and OS, 81%/82%/59%/42%, respectively (all *p* values <0.001). Notably, no significant differences were observed for cN+pN0 versus cN0pN0 (all *p* values >0.05; Figure 2, A–F). While the outcomes of patients with cN+pN0 disease were similar to those of cases with cN0pN0, they were more favorable than those observed in patients with cN0pN+/cN+pN+ disease. In contrast, the clinical outcomes of patients with cN0pN+ disease were better than those of patients with cN+pN+ disease (Figure 2, A–F) – which may be explained by a lower burden of pathological nodal disease. No cases of pN3a disease were identified. The frequencies of patients with pN1, pN2, and pN3b in those with cN0pN+ and cN+pN+ disease were 49%/34%/17% and 15%/23%/62%, respectively (*p* < 0.001).

### 3.3 Five‐year outcomes of patients with pN0 Disease stratified according to treatment modality

**FIGURE 3:**
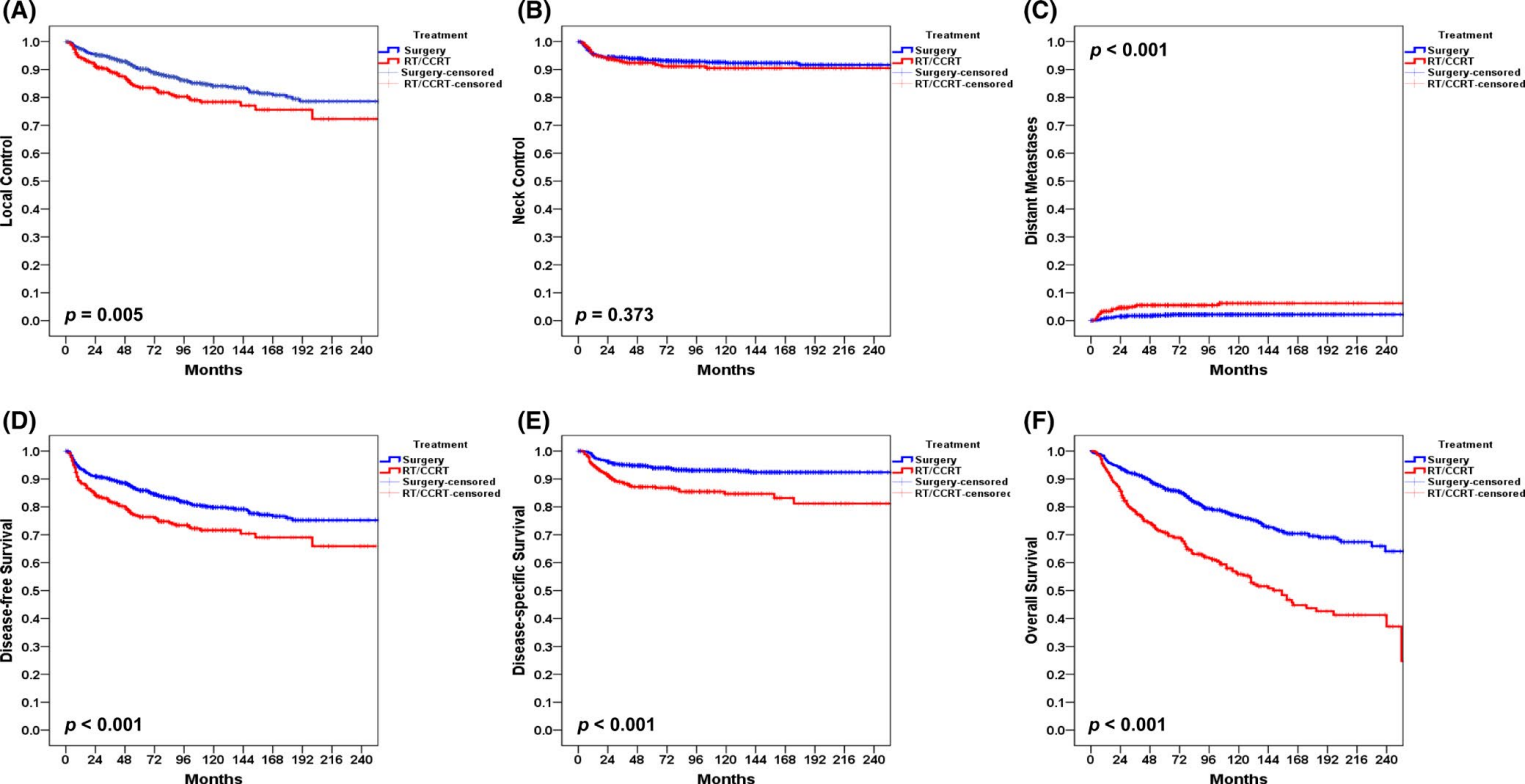
Kaplan–Meier plots of 5‐year local control (A), neck control (B), distant metastases (C), disease‐free survival (D), disease‐specific survival (E), and overall survival (F) for patients with pN0 disease treated with surgery alone and surgery plus adjuvant therapy

The 5‐year rates in patients with pN0 disease treated with surgery alone versus surgery plus adjuvant therapy were as follows: LC, 90% versus 83%, *p* = 0.005; NC, 93% versus 92%, *p* = 0.373; DM, 2% versus 5%, *p* < 0.001; DFS, 86% versus 76%, *p* < 0.001; DSS, 94% versus 87%, *p* < 0.001; and OS, 86% versus 71%, respectively (Figure 3, A–F, all *p* values <0.05, except for NC). Thus, the clinical outcomes of patients with pN0 disease treated with surgery plus adjuvant therapy were generally less favorable than those of cases who received surgery alone – the only exception being neck control.

### 3.4 Five‐year neck control rates in patients with pN0, cN0pN0, and cN+pN0 disease stratified according to planned and actual treatment

**FIGURE 4:**
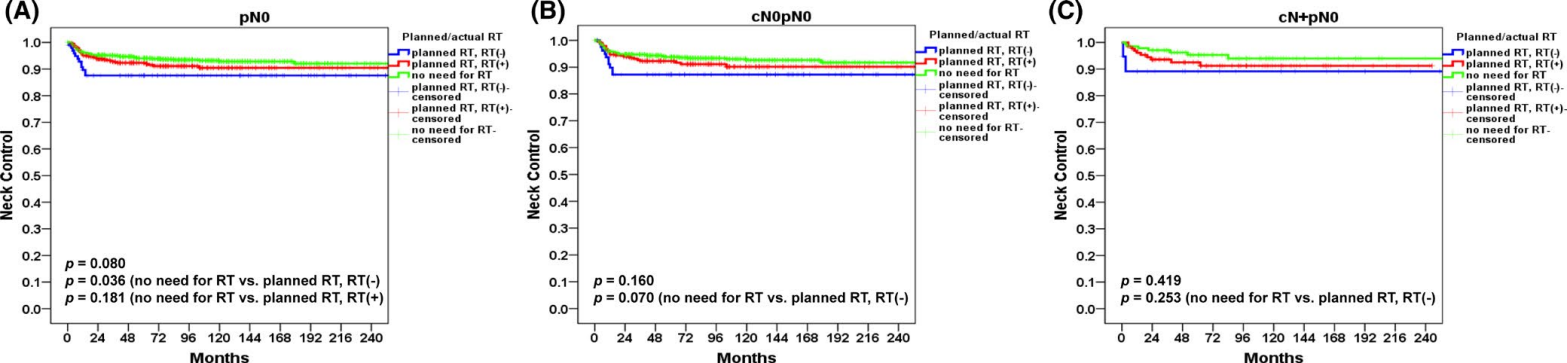
Kaplan–Meier plots of 5‐year neck control after stratification for no RT required, RT previously planned but not delivered, and RT given as previously planned in patients with pN0 disease (A), cN0pN0 disease (B), and cN+pN0 disease (C)

The 5‐year NC rates in patients with pN0 disease after stratification for planned surgery alone (*n* = 787), adjuvant therapy given as previously planned (*n* = 422), and adjuvant therapy (RT/CCRT) previously planned but not delivered (*n* = 100) were 94%, 92%, and 88%, respectively (*p* = 0.080). The NC control rate was higher in patients who received planned surgery alone compared with those in whom adjuvant therapy was previously planned but not delivered (94% vs. 88%, *p *= 0.036); however, the former group did not differ from patients in whom adjuvant therapy was given as previously planned (94% vs. 92%, *p* = 0.181; Figure 4A). The 5‐year NC rates in the cN0pN0 subgroup stratified as previously mentioned were 94%, 92%, and 87%, respectively (*p* = 0.160; planned surgery alone vs. adjuvant therapy previously planned but not delivered, *p* = 0.070; Figure 4B). The 5‐year NC rates in the cN+pN0 subgroup stratified as previously mentioned were 95%, 93%, and 89%, respectively (*p* = 0.419; planned surgery alone vs. adjuvant therapy previously planned but not delivered, *p* = 0.253; Figure 4C).

### 3.5 Multivariable analysis of 5‐year outcomes in patients with pN0 disease

We initially identified the following reference categories (HR = 1): cN0pN0, female sex, age <65 years, negative history for alcohol drinking, negative history for betel chewing, negative history for cigarette smoking, pT1−2 disease, well/moderate differentiation, DOI <10 mm, margin >4 mm, absence of perineural invasion, absence of lymphatic invasion, absence of vascular invasion, and treatment with surgery alone. MVA with a forward stepwise selection procedure identified the following risk factors as unfavorable independent risk factors for 5‐year outcomes: betel chewing (LC and DFS), poor differentiation (NC, DM, DFS, DSS, and OS), perineural invasion (NC, DFS, and DSS), lymphatic invasion (NC, DSS, and OS), DOI ≥10 mm (DM and DSS), pT3−4 disease (DFS and OS), and age ≥65 years (OS) (Table 2). Notably, cN+pN0 disease was not significantly associated with survival outcomes either in UVA (data not shown) or MVA.

**TABLE 2:** Multivariable analyses of risk factors for 5‐year local control, neck control, distant metastases, and survival rates in patients (*n* = 1309) with oral cavity cancer and pathologically negative nodes

Risk factor	Local control		Neck control		Distant metastases		Disease‐free survival		Disease‐specific survival		Overall survival	
	HR (95% CI)	*p*	HR (95% CI)	*p*	HR (95% CI)	*p*	HR (95% CI)	*p*	HR (95% CI)	*p*	HR (95% CI)	*p*
Betel chewing (*n* = 1069)	2.076 (1.306–3.300)	.002	—	ns	—	ns	1.434 (1.012–2.032)	0.043	—	ns	—	ns
Poor differentiation (*n* = 77)	—	ns	2.657 (1.491–4.736)	0.001	5.141 (2.450–10.792)	<0.001	2.134 (1.411–3.226)	<0.001	2.816 (1.642–4.829)	<0.001	1.572 (1.052–2.350)	0.027
Perineural invasion (*n* = 283)	—	ns	2.193 (1.442–3.334)	<0.001	—	ns	1.534 (1.168–2.014)	0.002	1.860 (1.251–2.764)	0.002	—	ns
Lymphatic invasion (*n* = 9)	—	ns	4.273 (1.044–17.497)	0.043	—	ns	—	ns	4.473 (1.095–18.282)	0.037	4.393 (1.793–10.764)	0.001
Depth of invasion ≥10 mm (*n* = 529)	—	ns	—	ns	3.095 (1.622–5.905)	0.001	—	ns	2.067 (1.403–3.047)	<0.001	—	ns
pT3–4 (*n* = 581)	—	ns	—	ns	—	ns	1.322 (1.032–1.693)	0.027	—	ns	1.674 (1.316–2.129)	<0.001
Age ≥65 (*n* = 189)	—	ns	—	ns	—	ns	—	ns	—	ns	1.612 (1.237–2.101)	<0.001
S+RT/CCRT (*n* = 422)	1.547 (1.155–2.073)	0.003		ns		ns		ns		ns	1.725 (1.362–2.184)	<0.001

Abbreviations: CCRT, chemoradiotherapy; CI, confidence interval; HR, hazard ratio; ns, not significant; RT, radiotherapy; S, surgery.

### 3.6 Subgroup analyses of cN+pN0 versus cN0pN0 disease after propensity score matching

**FIGURE 5:**
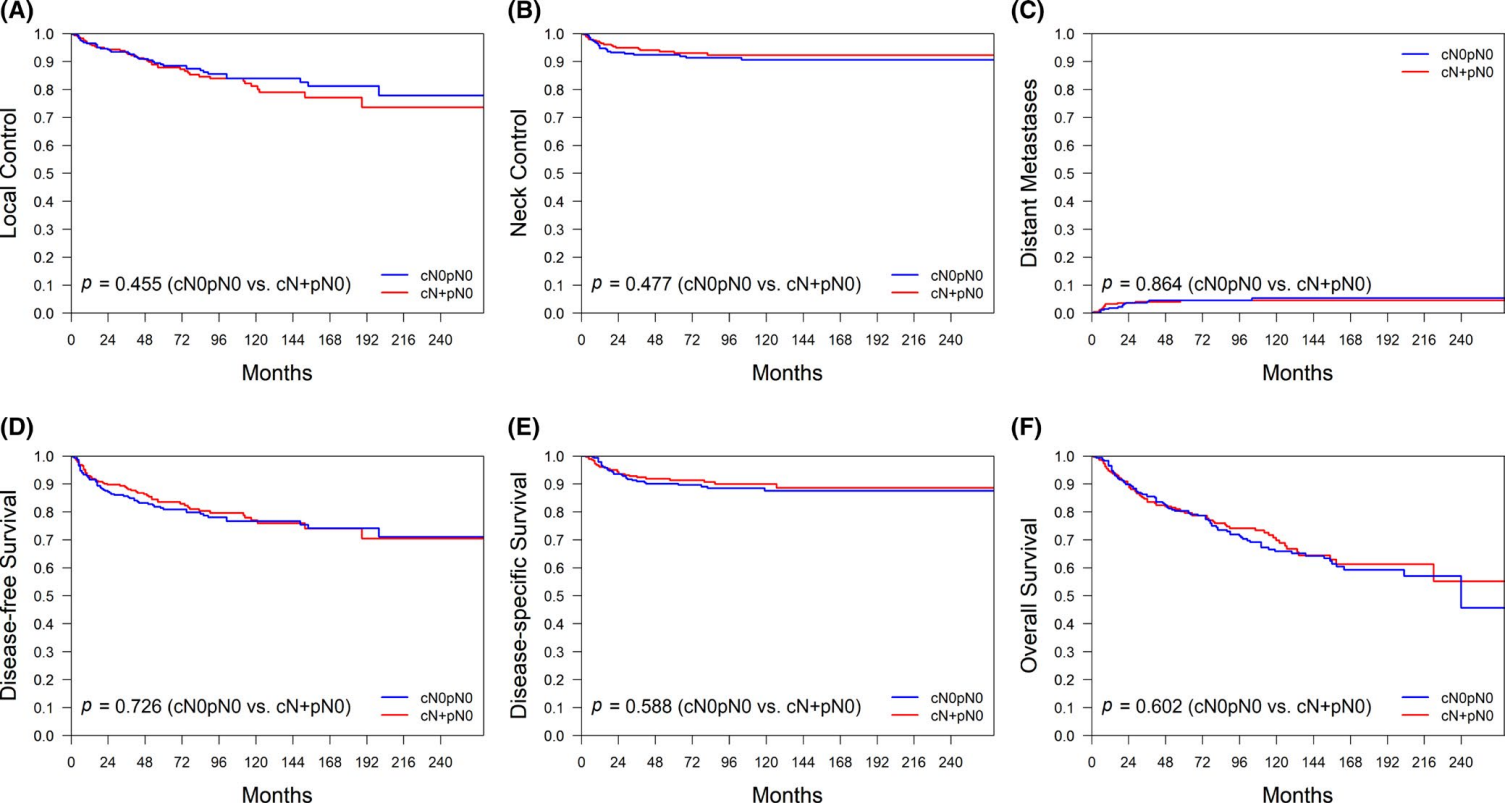
Kaplan–Meier plots of 5‐year local control (A), neck control (B), distant metastases (C), disease‐free survival (D), disease‐specific survival (E), and overall survival (F) for patients with cN0pN0, cN+pN0, cN0pN+, and cN+pN+ disease in the propensity score‐matched cohort

Owing to the baseline differences in terms of severity, we applied propensity score matching to obtain a matched cohort of patients with cN+pN0 and cN0pN0 disease (*n* = 284 each; Table 1). The results from this propensity score‐matched analysis did not appreciably differ compared with the original dataset with respect to local control, neck control, distant metastases, and survival figures (all *p* > 0.05; Figure 5A–F).

## 4 DISCUSSION

The clinical outcomes of patients with OCSCC are heavily influenced by the presence of nodal metastases. As expected, the 5‐year DSS and OS rates observed in our study were markedly less favorable for patients with pN+compared to this with pN0. Notably, patients with pN0 disease tended to relapse locally rather than regionally (Figure 1, Figure 2A, B, Figure 3A, B). Conversely, local, regional, and distant recurrences occurred more frequently in presence of pN+disease (Figure 1, Figure 2A–C). The results reported by Amit et al.^13^ demonstrated that cN+pN0 disease is an independent RF for patients with OCSCC. This can be explained by a suboptimal lymph node dissection and an inaccurate histopathological evaluation – which may in turn lead to understaging. However, their conclusions were based on the analysis of DSS and OS–without a specific focus on the relationship between cN+pN0 disease and NC. The results of our study demonstrate that cN+pN0 disease was not independently associated with reduced 5‐year LC, NC, DM, DFS, DSS, and OS rates. Taken together, these findings indicate that cN+pN0 disease is not an independent RF after adjustment for potential confounders in MVA.

Because untreated micrometastases are expected to evolve into overt metastases during the course of disease,^21^ selective ND is recommended in patients at high risk for occult metastases. Following selective ND of patients with pN0 disease, the possibility of missing microscopic nodal spread should still be considered.^22^ In this scenario, the concept of lymph node density–which is traditionally applied to pN+disease and expressed as the ratio between the number of pN+and the lymph node yield – may be extended to patients with pN0 disease and pathologically undetected micrometastases. This can be achieved by calculating the ratio between the number of nodes with undetected micrometastases and the lymph node yield. Under these circumstances, NC is expected to improve in parallel with the number of dissected nodes. On analyzing the two largest cohort studies published in the field (Table 3), Amit et al.^13^ reported a mean nodal yield of 29–which is significantly lower than that observed in our current report (mean in the entire cohort: 42; cN+pN0: 52; cN0pN0: 39).

**TABLE 3:** Published studies focusing on clinicopathological risk factors and prognosis of patients with oral cavity cancer and pathologically negative nodes

Authors (years of recruitment)	Tumor subsite/Number of patients/AJCC staging manual	Pathological variables (%)	Five‐year outcome/adjuvant therapy (%)/lymph node (LN) yield	Independent adverse risk factors (multivariable analysis)
Liao et al. (1996–2018), current study	Oral cavity/cN0pN0 (1019)cN+pN0 (290)/AJCC: 8th	pT (pT3–4, 44%), poor differentiation (6%), perineural invasion (22%), lymphatic invasion (1%), vascular invasion (1%), DOI (≥10 mm, 41%), margins (≤4 mm, 10%)	LC: 88% NC: 93% DM: 3% DFS: 83% DSS: 92% OS: 81%/adjuvant: 32%/mean: 42 LNs	LC: betel chewing NC: poor differentiation, perineural invasion, lymphatic invasion DM: poor differentiation, DOI ≥10 mm DFS: betel chewing, poor differentiation, perineural invasion, pT3–4 DSS: poor differentiation, perineural invasion, lymphatic invasion, DOI ≥10 mm OS: poor differentiation, lymphatic invasion, pT3–4, age ≥65 years
Amit et al.^13^ (1990–2011)	Oral cavity/cN0pN0 (1913)cN+pN0 (345)/AJCC: 6th	pT (pT3–4, 45%)	DSS: 81%,OS: 72%/adjuvant: 51%/mean: 29 LNs	DSS: cN, age ≥65 years, pT, DOI >4 mm, positive margins OS: cN, age ≥65 years, pT, LN yield ≤18 nodes, DOI >4 mm, positive margins
So et al.^9^ (1995–2016)	Tongue/pN0 (166)/AJCC: 7th	pT (pT3–4, 11%), perineural invasion (15%), lymphovascular invasion (12%), DOI (≥5 mm, 53%), margins (≤5 mm, 30%)	NC: 92% (3–year)/adjuvant: 25%/LNs: nr	NC: male sex
Cassidy et al.^10^ (2003–2013)	Tongue/cN0pN0 (112)cN0pNx (68)/AJCC: 7th	pT (pT3–4, 9%), poor differentiation (8%), perineural invasion (31%), lymphovascular invasion (20%), DOI (≥3 mm, 74%), margins (<5 mm, 32%)	LRC, OS: nr/adjuvant: 27%/LNs: nr	LRC: lymphovascular invasion, no elective ND, margins <5 mm OS: lymphovascular invasion, age >44 years
Chang et al.^11^ (2002–2015)	Oral cavity/pN0 (216)/AJCC: 8th	pT (pT3–4, 37%), perineural invasion (6%), lymphovascular invasion (2%), DOI (>5 mm, 62%), margins (≤5 mm, 26%)	OS: 74%/adjuvant: nr/LNs: nr	OS: DOI >5 mm, positive margins, perineural invasion, lymphovascular invasion
Chinn et al.^12^ (1998–2009)	Oral cavity/pN0 (88)/AJCC: nr	pT (pT3–4, 44%), perineural invasion (23%)	LRC, DFS, DSS, OS: nr/adjuvant: 40%/LNs: nr	LRC: perineural invasion, vascular invasion DFS: perineural invasion DSS: poor differentiation OS: no significant predictor identified

Abbreviations: DFS, disease‐free survival; DM, distant metastases; DOI, depth of invasion; DSS, disease‐specific survival; LC, local control; LN, lymph node; LRC, locoregional control; NC, neck control; ND, neck dissection; nr, not reported; OS, overall survival.

The accuracy of the pN0 diagnosis is clearly dependent on a sufficiently extensive lymph node harvesting during ND–resulting in a high cervical node yield. The higher nodal yield in our patients with cN+pN0 disease may at least in part explain the lack of significant differences in terms of NC when compared with cN0pN0 disease. While the prevalence of pT3‐4 disease in our report was in line with the study by Amit et al.^13^ (44% vs. 45%, respectively), the use of adjuvant therapy was less frequent in our cohort (32% vs. 51%, respectively). This can be explained by the adoption of the CGMH guidelines (instead of the NCCN recommendations) as of 2008.^7^ We have previously shown that–compared with the NCCN recommendations–the CGMH guidelines can reduce by 28% the number of intermediate‐risk patients that should receive RT, without compromising 5‐year DSS and OS rates (94% and 87%, respectively). Notably, the 5‐year DSS and OS rates observed in our study were not inferior to those reported by Amit et al.^13^ (92%/81 vs. 81%/72%, respectively).

The clinical outcomes of patients with pN0 disease may be dependent on the presence of adverse clinicopathological RFs – which can in turn influence the decision to implement adjuvant therapy. In published studies focusing on pN0 disease (Table 3), the prevalence rates of pT3−4 disease, perineural invasion, and the use of adjuvant therapy were 9−45% (44% in our study), 6−31% (22% in our study), and 25−51% (32% in our study), respectively. Specific adverse prognostic factors are not uniform in the available literature, and these discrepancies may be explained by differences in the definition of the study variables or clinical endpoints. For example, lymphovascular invasion was regarded by the NCCN guidelines as an adverse prognostic factor until 2018; subsequently, the presence of lymphatic and vascular invasion was considered separately. The results of our study indicate that lymphatic invasion – rather than vascular invasion – were an independent adverse prognostic factor for NC, DSS, and OS. While some of the published studies considered locoregional control as an endpoint of interest, it was not specified whether the events used for outcome definition occurred locally or regionally. In the current study, we observed a high 5‐year NC rate (93%) – which is in line with the 3‐year NC rate reported by So et al.^9^ (92%; Table 3). While there were three adverse pathologic RFs for NC in our study (poor differentiation, perineural invasion, and lymphatic invasion), So et al.^9^ identified male sex as the only unfavorable prognostic factor for the same endpoint. A potential explanation for the conflicting findings may be related to the higher NC rate (which reflects a lower number of neck recurrences) occurring in a more limited sample size (*n* = 166).

When pathological evidence of neck nodal metastases is lacking, the neck basin is frequently excluded from the RT field. While the presence of specific pathological RFs poses an indication for RT/CCRT to the primary tumor site, the elective irradiation of the neck in patients with pN0 disease remains a matter of ongoing debate – especially at the contralateral side. In a phase 2 study, Contreras et al. reported an unirradiated neck control rate of 97% for resected SCC of the head and neck (*n* = 72; OCSCC [*n* = 14], other subsites [*n* = 58]). Notably, no patient had contralateral neck received irradiation, and in 17 patients (24%), only the primary site was treated. Treatment failures occurred only in two patients with OCSCC and pN0 disease who did not receive neck irradiation.^23^ It is thus possible that patients with OCSCC are at an increased risk of neck failure compared with those having head and neck malignancies originating from other anatomical subsites.

Because intensive adjuvant treatment may result in significant morbidity, the question as to whether the benefits of CT/CCRT outweigh its risks in pN0 disease remains unanswered. In this regard, an analysis of six studies conducted in 325 patients did not show a significant benefit (*p* = 0.059) of RT guided by the presence of RFs in terms of 5‐year OS.^24^ In our study, we observed that – compared with patients who underwent planned surgery alone – those in whom adjuvant therapy was previously planned (because of the presence of RFs) but not delivered had a significantly less favorable 5‐year NC rate (94% vs. 88%, respectively, *p* = 0.036). However, the former group (5‐year NC rate: 94%) did not differ significantly (*p* = 0.181) from patients in whom adjuvant therapy was given as previously planned (5‐year NC rate: 92%). These results suggest that adjuvant therapy may actually improve NC.

Despite a higher burden of risk factors, the prognosis of patients with cN+pN0 disease did not differ from that observed in cases with cN0pN0. The higher nodal yield and the more frequent use of adjuvant therapy in patients with cN+pN0 disease may explain the lack of significant outcome differences compared with those with cN0pN0 disease. However, we found that certain baseline RFs – including betel quid chewing, pT status, depth of invasion, perineural invasion, and postoperative treatment modalities – were not well balanced in the two study groups (cN+pN0 and cN0pN0). To account for the potential confounding impact of these variables, we applied propensity score matching and devised a matched data set of patients with cN+pN0 and cN0pN0 disease. However, the results obtained in the propensity score‐matched cohort did not differ significantly from those observed in the original data set (Figure 5A–D). Collectively, these findings indicate that our main conclusions regarding the prognostic significance of cN+pN0 are not significantly affected by the observed baseline differences.

There are limitations to the current study. First, its single‐center design may have limited the external validity of the results. Second, the retrospective nature of the research could be associated with information bias, and approximately 20% of the study patients harboring RFs did not receive adjuvant therapy. Finally, all participants were uniformly treated with surgery −either with or without adjuvant therapy. More studies are necessary to confirm our findings and to identify a specific subgroup of patients with pN0 disease who will most likely benefit from adjuvant therapy.

In conclusion, our study demonstrates that patients with OCSCC and cN+pN0 disease are characterized by a higher prevalence of adverse RFs. However, this was not found to translate into a less favorable prognosis – probably because of a sufficient number of excised nodes and an optimal selection of candidates for adjuvant therapy. Additionally, patients with cN+pN0 disease had a higher 5‐year NC rate – especially in the subgroups of those who underwent planned surgery alone or adjuvant therapy as previously planned (93−95%). Because the prognosis of patients with cN+pN0 disease did not differ from that of cases with cN0pN0, the presence of cN+pN0 disease should not be considered as an adverse prognostic factor in patients with OCSCC.

## CONFLICT OF INTEREST

The authors declare no conflicts of interest.
